# Notes and News

**Published:** 1899-03-04

**Authors:** 


### Notes and News.

(Collected and Communicated.)

### HOSPITAL MEETINGS.

University College Hospital.—Annual Meeting, Friday, March 24th, 4 p.m.

City of London Hospital for Diseases of the Chest, Victoria Park.—Annual Meeting, Monday, March 6th, at the Gresham College, Basinghall Street, at 4 p.m.

The staff under the Sanitary Inspector at Aberdeen are kept very busy at present, owing to an epidemic of measles. Last week upwards of 600 cases were reported, entailing inspection and disinfection.

Mr. Alfred Cooper, F.R.C.S., has been elected honorary consulting physician to St. Mark's Hospital, City Road. Mr. Cooper has been on the staff of the hospital for 34 years.

The death is announced of Professor Rutherford, Professor of Physiology, at Edinburgh. Professor Rutherford was sixty years of age. He was a brilliant lecturer, and will be much missed in scientific and social circles.

The public and also local authorities have taken up the subject of stamping out tuberculosis with energy, but it does not appear that the Government is yet inclined to co-operate very heartily, In reply to Mr. Warner's question in the House of Commons as to the intentions of the Government in respect to the inspection of tuberculous cattle, he was informed that the Government did not intend to appoint inspectors, nor had any proposals been formulated as to the powers to be granted to local authorities to carry out inspection.

The scheme to establish schools for the study of tropical diseases both in London and Liverpool is receiving practical encouragement by those who know how greatly knowledge in this direction will be of practical benefit to British subjects abroad. Mr. Chamberlain is to preside at the festival dinner to be held in connection with the formation of the school at the Albert Dock Hospital in May, and doubtless the movement will then receive a fresh impetus, and practical support and valuable testimony as to the value of the scheme will be forthcoming.

The French Congress of Medicine will be held this year at Lille, and will open on July 28th. An International Congress of the Medical Press will be held in Paris in 1900.

Lord Egerton of Tatton has been nominated as Chancellor of the Order of the Hospital of St. John of Jerusalem by H.R.H. the Prince of Wales, Grand Prior of the Order.

St. Thomas's Hospital has received a donation of £1,000 from "G. R." for the endowment of a surgical bed, "In Memoriam," being the fourth bed endowed by the same generous benefactor.

The Pope, whose illness is considered of an alarming nature, is under the care of Professors Lapponi and Mazzeni at the Vatican. The accounts of the nature of the illness from which the Pope is suffering are conflicting.

The effect of the new regulations of the Royal Army Medical Corps are markedly shown by the candidature at the recent examination. For the first time for some years past candidates have largely exceeded vacancies in number.

The new laboratories in connection with the Middlesex Hospital Medical School, we understand, are now completed, and are equipped with all the best modern appliances for the purpose of instruction and original research. A conversazione will be held on the evening of March 15th in the new buildings, when many objects of interest will be exhibited.

Through the kindness of the Rev. Sydney Vatcher, vicar of St. Phillip's, Stepney, the London Hospital is to have the use of a portion of the churchyard as an "airing ground." The churchyard is in close proximity to the hospital, and direct communication is now being arranged. The congregation of St. Phillip's has always been augmented from the hospital staff, and if the church is in direct communication with the hospital it is possible it may be utilised in place of the chapel.

A sessional meeting of the Sanitary Institute will be held at the Parkes Museum on Wednesday, March 8th, at eight p.m., when a discussion will be opened on "The Establishment of Public Abattoirs in the Metropolis in Relation to the Prevention of Tuberculosis," by William Arthur Bond, M.A., M.D., B.Sc. Camb., D.P.H., M.O.H. Holborn.

Guy's Hospital has received the munificent donation of £5,000 from Sir F. Wills to further education at the medical school. It is to be hoped that Mr. Balfour's appeal on behalf of medical research may be responded to by other generous persons, for it is evident that the advancement of medical science cannot be secured without a certain latitude of expenditure, and it is equally accepted that the expenses of medical schools should not be defrayed out of funds given by the public for the treatment of the sick. The necessity for special endowment is self-evident, and medical research is just as worthy of encouragement as any form of learning pursued at our universities.

